# Towards resolving the complex paramagnetic nuclear magnetic resonance (NMR) spectrum of small laccase: assignments of resonances to residue-specific nuclei

**DOI:** 10.5194/mr-2-15-2021

**Published:** 2021-01-29

**Authors:** Rubin Dasgupta, Karthick B. S. S. Gupta, Huub J. M. de Groot, Marcellus Ubbink

**Affiliations:** Leiden Institute of Chemistry, University of Leiden, Gorlaeus Laboratory, Einsteinweg 55, 2333 CC, Leiden, the Netherlands

## Abstract

Laccases efficiently reduce dioxygen to water in an active site containing a tri-nuclear copper centre (TNC). The dynamics of the protein matrix is a determining factor in the efficiency in catalysis. To probe mobility, nuclear magnetic resonance (NMR) spectroscopy is highly suitable. However, several factors complicate the assignment of resonances to active site nuclei in laccases. The paramagnetic nature causes large shifts and line broadening. Furthermore, the presence of slow chemical exchange processes of the imidazole rings of copper ligand results in peak doubling. A third complicating factor is that the enzyme occurs in two states, the native intermediate (NI) and resting oxidized (RO) states, with different paramagnetic properties. The present study aims at resolving the complex paramagnetic NMR spectra of the TNC of *Streptomyces coelicolor* small laccase (SLAC). With a combination of paramagnetically tailored NMR experiments, all eight His N δ 1 and H δ 1 resonances for the NI state are identified, as well as His H β protons for the RO state. With the help of second-shell mutagenesis, selective resonances are tentatively assigned to the histidine ligands of the copper in the type-2 site. This study demonstrates the utility of the approaches used for the sequence-specific assignment of the paramagnetic NMR spectra of ligands in the TNC that ultimately may lead to a description of the underlying motion.

## 1 Introduction

**Scheme 1:**
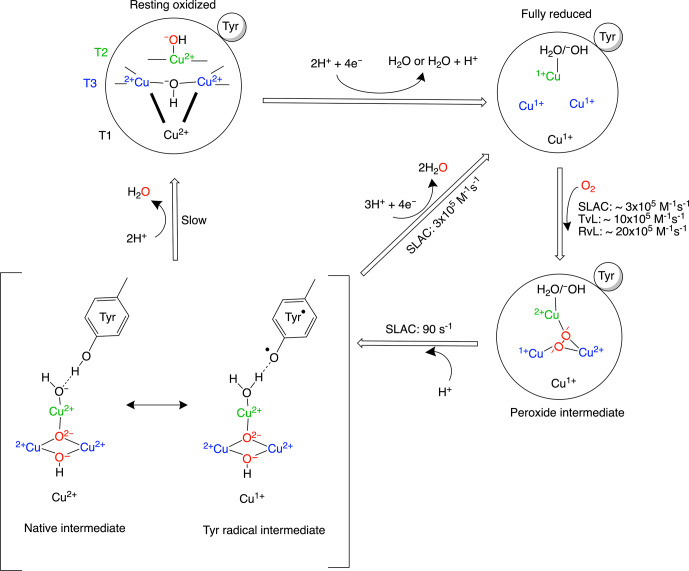
Reaction mechanism of the oxygen reduction reaction in SLAC. The coordination of the copper ions in the TNC is shown in the resting oxidized state. The T3 copper ions (blue) are coordinated to three histidine N ε 2 atoms and the hydroxyl group. The two histidines from the HCH motif connecting the T1 site with the T3 site are shown as bold black lines. The T2 copper (green) is coordinated to two histidine N ε 2 atoms and a water/hydroxide ligand. The rates for oxygen binding are shown for laccases from several organisms, SLAC from *S. coelicolor*, TvL from *Trametes versicolor* laccase and RvL for *Rhus vernicifera* laccase (Heppner et al., 2014). An intermediate for SLAC is shown with the Tyr ${}^{⚫}$ radical (Gupta et al., 2012; Tepper et al., 2009). This intermediate has only been reported for SLAC and hCp (Tian et al., 2020).

Multicopper oxidases (MCOs) oxidize a wide variety of substrates at their type-1 (T1) site and catalyse the four-electron reduction of molecular oxygen to water at the tri-nuclear copper centre (TNC). The TNC consists of a type-2 (T2) copper site and a binuclear type-3 (T3) copper site. Based on crystallographic, spectroscopic and theoretical studies, the present model of the oxygen reduction mechanism by the TNC is shown in Scheme 1 (Gupta et al., 2012; Heppner et al., 2014; Quintanar et al., 2005b; Tepper et al., 2009; Yoon and Solomon, 2007). The two-domain small laccase from *Streptomyces coelicolor* (SLAC) has been reported to involve the formation of a tyrosine radical (Tyr108 ${}^{⚫}$ ) near the T2 site during the peroxide intermediate (PI) to native intermediate (NI) conversion (Gupta et al., 2012; Tepper et al., 2009). This radical has been suggested to act as protection against the reactive oxygen species (ROS) that can be formed due to the long-lived peroxide intermediate state (Gupta et al., 2012; Kielb et al., 2020). The tyrosyl radical was shown to be reduced by the protein environment via tryptophan and tyrosine residues around the T2 site (Kielb et al., 2020). A similar role was proposed for Tyr107 in human ceruloplasmin (hCp). hCp is a ferroxidase critical for iron homeostasis. It oxidizes Fe ${}^{2+}$ to Fe ${}^{3+}$ for iron transport. In serum the hCp is active under low-Fe ${}^{2+}$ and high-O ${}_{2}$ concentration. This leads to a partially reduced intermediate that can form ROS. The tyrosine radical protects the protein from this partially reduced state (Tian et al., 2020).

Although the reaction mechanism of laccase is well characterized, information about motions around the TNC is limited. The oxygen-reduction process is a multi-step reaction involving transfer of four electrons and protons with oxidation and reduction of the copper ions (Scheme 1). Each step is associated with its respective activation energy barrier, and the motions of the protein, especially within the active site, may be useful in reduction or crossing of these barriers. Such motions have been reported for many proteins, for example dihydrofolate reductase, adenylate kinase and cytochrome P450 (Hammes-Schiffer, 2006; Hammes-Schiffer and Benkovic, 2006; Henzler-Wildman et al., 2007; Poulos, 2003). Characterization of motion at the TNC of laccase can help in designing a functional framework for understanding the natural process and the de novo design of efficient bioinspired catalysts. Three or more independent chemical exchange processes, tentatively assigned to the coordinating histidine residues at the TNC, were reported using paramagnetic nuclear magnetic resonance (NMR) spectroscopy on the T1 copper-depleted variant of SLAC, SLAC-T1D (Dasgupta et al., 2020a). However, further characterization of motions requires assignments of the NMR resonances very near to the TNC. The paramagnetic nature of the copper ions causes broadening and chemical shifts outside of the diamagnetic envelope, making it impossible to employ standard multidimensional protein assignment experiments. Assignment is further complicated by two reasons. SLAC spectra are a mixture of the RO and NI states (Scheme 1) (Machczynski and Babicz, 2016). In the RO state the T2 Cu ${}^{2+}$ is isolated, causing broadening of the signals of nearby proton spins beyond detection. The two copper ions in the T3 site are antiferromagnetically coupled, with a low-lying triplet state that is populated at room temperature, causing paramagnetically shifted (in the range of 12–22 ppm), detectable resonances of nearby protons. In the NI state all the copper ions are coupled, resulting in a frustrated spin system, with strongly shifted ( > 22 ppm) but observable resonances (Zaballa et al., 2010). The second cause of complexity is that the mentioned exchange processes of the coordinating histidine residues result in peak doublings because the exchange rates are in the slow exchange regime relative to the resonance frequency differences. In this study, we aimed to resolve further this complicated paramagnetic NMR spectrum. Using differently labelled samples and tailored HMQC experiments, the presence of all eight-histidine ligand N δ 1 and H δ 1 resonances in the NI state could be established. The first studies of the RO state identified resonances as histidine H δ 1 or H β protons and a second coordination shell mutant allowed for the first residue and sequence-specific assignment. The study demonstrates the utility of the approaches used for the sequence-specific assignments of the ligands in the TNC that may ultimately lead to a description of the underlying motions.

## 2 Results and discussion

### 2.1 Identification of nitrogen-attached protons in the NI state

**Figure 1:**
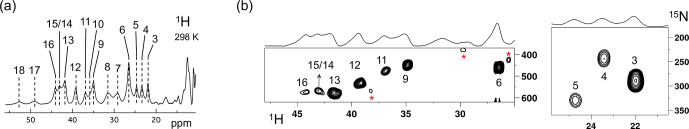
SLAC-T1D NMR spectra at 298 K. **(a)** 1D ${}^{1}$ H WEFT spectrum of SLAC-T1D and **(b)** ${}^{15}$ N- ${}^{1}$ H HMQC spectra of ${}^{15}$ N-His perdeuterated SLAC-T1D in a back-exchanged environment. The numbering is adopted from Dasgupta et al. (2020a). Noise peaks in the spectrum are marked with a red asterisk. The 1D ${}^{1}$ H WEFT spectrum is shown above the HMQC spectrum.

**Figure 2:**
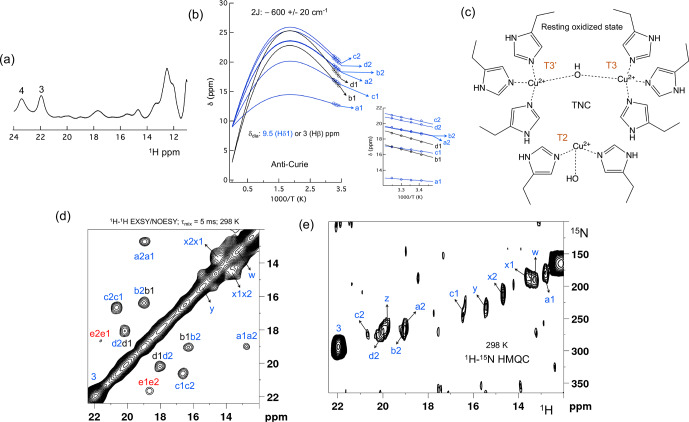
The spectral region of the RO state. **(a)** 1D ${}^{1}$ H spectra of the RO region from Fig. 1a. Resonances 3 and 4 of the NI state are shown for comparison; **(b)** temperature dependence of the chemical shift for resonances a1, a2, b1, b2, c1, c2, d1 and d2, fitted to the two-metal centre model (Eq. S1). The inset shows the experimental region of the fit. The corresponding hyperfine coupling constants are given in Table S5 of the Supplement; **(c)** schematic representation of the RO state of the TNC. The T3 and T2 copper ions are marked. **(d)** ${}^{1}$ H– ${}^{1}$ H EXSY/NOESY spectra of SLAC-T1D for the region between 12 and 22 ppm; **(e)** ${}^{15}$ N- ${}^{1}$ H HMQC spectra of the ${}^{15}$ N uniformly labelled SLAC-T1D (12 to 22 ppm in the ${}^{1}$ H dimension). The resonances marked in blue are for the nitrogen-attached protons, while resonances in black are for carbon-attached protons. Resonances in red in panel **(d)** could not be assigned to either nitrogen-linked or carbon-linked protons due to a low S/N ratio.

The Fermi contact shifted resonances for SLAC-T1D were reported before and here we use the numbering used in our previous study (Dasgupta et al., 2020a; Machczynski and Babicz, 2016). Eighteen resonances were found between 15 and 60 ppm. Resonances 1 and 2 were assigned to a region that is attributed to the RO state; therefore, we followed the numbering from 3 to 18 in the present work (Fig. 1a). Resonance 10 is from a proton bound to carbon and overlaps with resonances 9 and 11 at temperatures > 293 K (Dasgupta et al., 2020a) (Fig. 1a). The ${}^{1}$ H resonances that exhibited exchange processes (3–5, 9–11 and 13–12) were assigned to H δ 1 nuclei from histidine coordinated to the copper ion (Dasgupta et al., 2020a). To verify this assignment, a paramagnetically tailored ${}^{1}$ H– ${}^{15}$ N HMQC experiment (Fig. S2 in the Supplement) was performed on a SLAC-T1D sample that was specifically labelled with ${}^{15}$ N histidine in a perdeuterated, back-exchanged environment. The evolution period was shortened to 500 µ s, balancing the time required for formation of antiphase magnetization and paramagnetic relaxation, to optimize the S/N ratio for most of the resonances (Ciofi-Baffoni et al., 2014; Gelis et al., 2003). A total of 10 resonances (3, 4, 5, 6, 9, 11, 12, 13, 14/15, 16; see Fig. 1b) were observed at ${}^{1}$ H chemical shifts of > 22 ppm. Resonances 7, 8 and 10 were not observed in this experiment, which is consistent with their assignment to carbon-attached protons (Dasgupta et al., 2020a). These results show unequivocally that the HMQC resonances derive from the H δ 1 protons of the coordinating histidine residues of the TNC, because only these protons are nitrogen-attached and close enough to experience such large paramagnetic shifts. The three pairs or resonances representing exchange processes (3–5, 9–11 and 13–12) are thus also from H δ 1 protons, in line with the suggested histidine ring motion being the involved chemical exchange process. The HMQC spectrum of uniformly ${}^{15}$ N labelled SLAC-T1D is similar to the ${}^{15}$ N-His specifically labelled the SLAC-T1D sample in a perdeuterated back-exchanged environment (data not shown for the ${}^{1}$ H resonances > 22 ppm but shown for the region 12 to 22 ppm; see Fig. 2b).

The relative intensities of signals in the range 22 to 55 ppm compared to those between 12 and 22 ppm show that SLAC-T1D is predominantly in the NI state (Figs. 1 and 2). In the NI state the T2 and the T3 sites are coupled, increasing the electronic relaxation rates of the unpaired electron spin S and thus reducing the paramagnetic relaxation rates of the nuclear spins. Therefore, it is expected that all eight ligand histidine residues are observable. In the ${}^{1}$ H– ${}^{15}$ N HMQC 10 resonances are seen, among which 3 undergo chemical exchange resulting in the observation of seven N δ 1–H δ 1 groups. Resonances 17 and 18 have exchange cross-peaks with resonances 15/14 and 16, respectively, at high temperatures (303 and 308 K) and a short mixing time in an EXSY/NOESY experiment (1 and 2 ms) (Dasgupta et al., 2020a). At temperatures of 298 K and higher, resonances 14 and 15 overlap (Fig. 1a) (Dasgupta et al., 2020a). Resonances 16 and 18 thus form a fourth exchange pair and the eighth histidine N δ 1–H δ 1 group can be attributed to the exchange pair of resonance 17 with either 14 or 15 (Dasgupta et al., 2020a). Due to the overlapping of resonances 14 and 15 at 298 K, they are not observed distinctly in ${}^{1}$ H– ${}^{15}$ N HMQC spectra (Fig. 1b). In conclusion, all eight H δ 1 from the coordinating histidines of the TNC in SLAC-T1D for the NI state are identified in the spectral region > 22 ppm, and five of them show peak doubling due to slow exchange.

### 2.2 Analysis of the RO state

Machczynski and Babicz (2016) reported that the signals in the spectral region between 12 and 22 ppm derive from the RO state (Fig. 2a), whereas the resonances > 22 ppm are attributed to the NI state (Machczynski and Babicz, 2016). In the RO state, the T2 copper is decoupled from the T3 site, resulting in a decrease in its electronic relaxation rate (Bertini et al., 2017). This effect broadens the resonances of nearby proton spins beyond detection for the T2 site ligands. In the RO state, the T3 copper ions are antiferromagnetically coupled and thus diamagnetic at low temperature (Bertini et al., 2017). At ambient temperature, the low-lying state with S = 1 is populated, resulting in paramagnetic shifts of the ligand protons (Bertini et al., 2017). The strong coupling via a hydroxyl moeity of the electron spins causes fast electronic relaxation and thus observable nuclear resonances for T3 ligands. T3 site ligands usually exhibit an anti-Curie behaviour; i.e. the chemical shift increases with an increase in temperature (Banci et al., 1990; Bertini et al., 1993; Bubacco et al., 2000; Tepper et al., 2006).

All the resonances in the 12 to 22 ppm region of SLAC-T1D in a ${}^{1}$ H– ${}^{1}$ H EXSY/NOESY spectrum display anti-Curie behaviour, suggesting that indeed they derive from histidine protons of the T3 site (Fig. 2d). Comparing the ${}^{1}$ H– ${}^{15}$ N HMQC and the ${}^{1}$ H– ${}^{1}$ H EXSY/NOESY of the ${}^{15}$ N uniformly labelled sample in this region, resonances a1, a2, b2, c1, c2, d2, x1, x2, y, z and w are nitrogen-linked protons (Fig. 2). The RO state is the minor state in SLAC-T1D, so the S/N ratio for the HMQC resonances is low. For comparison, resonance 3, which belongs to the NI state (Fig. 2e), is shown as well. ${}^{1}$ H resonances e1 and e2 could not be assigned to either carbon- or nitrogen-linked protons due to their low S/N ratio.

Using a two-metal centre model to calculate the singlet-triplet energy gap (2J) from the temperature dependence of the chemical shifts (Eq. S1), a 2J value of - 600 ± 20 cm ${}^{-1}$ was obtained, within the range of the previous reported values ( - 550 to - 620 cm ${}^{-1}$ ) for the RO state of laccase (Fig. 2c) (Battistuzzi et al., 2003; Machczynski and Babicz, 2016; Quintanar et al., 2005b). It is assumed that resonances a1, a2, b2, c1, c2, and d2 (only isolated resonances were selected) are the Fermi contact shifted resonances of the H δ 1 of the coordinating histidine residues at the T3 site in the RO state, as supported by their presence in the HMQC spectrum (Fig. 2). The diamagnetic chemical shift for these resonances was set to 9.5 ppm (BMRB – Biological Magnetic Resonance Bank – average for histidine ring H δ 1) (Zaballa et al., 2010). To establish the diamagnetic chemical shifts of resonances b1 and d1, which are not nitrogen-attached, the 2J coupling strength was then fixed to - 600 cm ${}^{-1}$ and the diamagnetic chemical shift was fitted and found to be 3.0 ± 0.5 ppm. This value strongly suggests that these resonances are from the β protons of coordinating histidines (the BMRB average for histidine H β is 3.1 ppm).

Since the temperature dependence of the cross-peak intensities as measured by their peak volume did not show a conclusive increasing trend with increase in temperature, we assumed them to be NOE- rather than EXSY-derived cross-peaks (Dasgupta et al., 2020a). Therefore, the cross-peaks of resonances b1–b2 and d1–d2 can be attributed to a NOE between the H δ 1 and H β proton of a histidine ligand. The cross-peaks between c1–c2 and a1–a2 appear to be NOE signals from nitrogen-attached protons (Fig. 2). The H δ 1 protons of the different histidine residues are not near, so it remains unclear from which spins these peaks derive. For resonances x1, x2, y, z and w (Fig. 2a), the analysis of the temperature dependence of the chemical shift was not possible due to the overlap.

### 2.3 Second-shell mutagenesis to assist assignments

To aid in the assignment of the paramagnetic spectrum, mutagenesis could be employed. However, mutation of histidine ligands is expected to result in loss of copper or at least in a severe redistribution of unpaired electron density, changing the chemical shifts of all paramagnetically shifted protons. In contrast, mutations in the second coordination sphere, of residues that interact with the coordinating ligands, may have moderate effects on the electron spin density distribution. One such mutant, Y108F, has been reported before (Gupta et al., 2012). Tyr108 interacts with the TNC in two ways, with the T2 site through the water/hydroxide ligand and with the T3 ligand His104 through the hydrogen bonding network involving Asp259 (Fig. S3a). Asp259 is conserved in all laccases, whereas Tyr108 is conserved in the two-domain laccases (Fig. S3b). Asp259 has been reported to play a role in modulating the proton relay during the oxygen reduction reaction (Quintanar et al., 2005a, p. 94), and it may also stabilize the Tyr108–TNC interaction.

**Figure 3:**
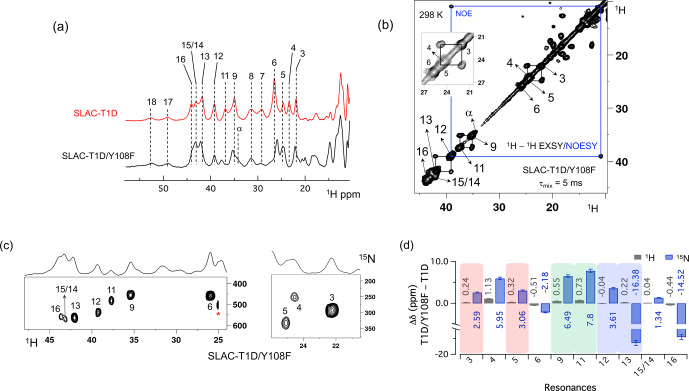
Spectra of SLAC-T1D/Y108F. **(a)** Comparison between the 1D ${}^{1}$ H WEFT spectrum of SLAC-T1D (red) and SLAC-T1D/Y108F (black). The numbering is shown for SLAC-T1D and is adopted from Dasgupta et al. (2020a); **(b)** ${}^{1}$ H– ${}^{1}$ H EXSY/NOESY of SLAC-T1D/Y108F at 298 K with a mixing time of 5 ms. NOE cross-peaks are connected with a blue rectangle. The remaining cross-peaks are exchange peaks. This distinction is based on the temperature-dependent profile of the integral volume of the cross-peaks as explained in Dasgupta et al. (2020a). The inset shows that the exchange cross-peaks are between 3 and 5. Resonance 4 partly overlaps with 5; **(c)** ${}^{1}$ H– ${}^{15}$ N HMQC spectra of ${}^{15}$ N uniformly labelled SLAC-T1D/Y108F. **(d)** The chemical shift changes ( Δδ ) between SLAC-T1D/Y108F and SLAC-T1D for the ${}^{1}$ H (black) and ${}^{15}$ N (blue). The error bars represent the standard deviation in the determination of the chemical shift. The three pairs of resonances displaying chemical exchange are highlighted by equal background colours. Positive (negative) values represent shift to the downfield (upfield) ppm for SLAC-T1D/Y108F.

The 1D ${}^{1}$ H WEFT (Bertini et al., 1993; Patt and Sykes, 1972) spectrum of SLAC-T1D/Y108F is similar to that of SLAC-T1D, suggesting that the variant SLAC is also predominantly in the NI state (Fig. 2a). Some changes in the chemical shift are present. Due to the Y108F mutation many of the ${}^{1}$ H resonances > 22 ppm are downfield shifted. Resonances 6 and 16 are upfield shifted and resonances 7, 8, 17 and 18 show no chemical shift change compared to SLAC-T1D (Fig. 3 and Table S2). Also, a new resonance α is observed. The HMQC spectrum in the region > 22 ppm of the ${}^{1}$ H is very similar to that of SLAC-T1D, in agreement with the ${}^{1}$ H WEFT spectrum (Fig. 3). Most of the ${}^{15}$ N resonances (3, 4, 5, 9, 11, 12 and 15) are downfield shifted, except resonances 6, 13 and 16, which are upfield shifted (Fig. 3 and Table S2). The three independent chemical exchange processes that were reported for the TNC of SLAC-T1D involving resonance pairs of 3–5, 9–11 and 13–12 (Dasgupta et al., 2020a) are conserved and the rates are not affected by the Y108F mutation (Table S1, Figs. 3b and S1c), suggesting that the phenolic OH group of Y108 is not involved in the chemical exchange process. The chemical shift changes show that the two states represented by 3–5 and 9–11, respectively, are affected similarly by the Y108F mutation (Fig. 3d). In contrast, the two states represented by resonance pair 13–12 are affected differently, because the nitrogen chemical shift is downfield shifted for resonance 12 and upfield shifted for resonance 13 (Fig. 3d).

**Figure 4:**
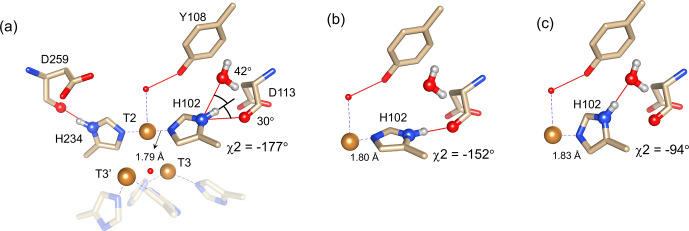
Alternative hydrogen bond acceptors for His102. **(a)** T2 site histidine ligands showing the hydrogen bonds for the N δ 1–H δ 1 groups. Protons were modelled using the algorithm as implemented in UCSF Chimera (Pettersen et al., 2004). His104 and H236 from the T3 ${}^{\prime }$ and T3 sites, respectively, are omitted for clarity. Hydrogen bonds are shown as red lines. The χ 2 dihedral angle and distance between His102 N ε 2 and the T2 copper are indicated. Also, the values for the angles [Asp113 CO - His102 N δ 1 - His102 H δ 1] and [water O628 - His102 N δ 1 - His102 H δ 1] are indicated. Ring rotation brings the H δ 1 in optimal position for hydrogen bond formation with either the Asp113 CO **(b)** or the water **(c)**. The new χ 2 dihedral angles and the corresponding His102 N ε 2–T2 copper distances are indicated.

It is proposed that resonances 13 and 16, which are most affected by the Y108F mutation (Fig. 3d), are from the histidine ligands of the T2 copper. Due to the proximity of the T2 copper and strong hydrogen bond to a water or hydroxide ligand, the electron spin density can be expected to be delocalized to the tyrosine ring. The loss of the hydrogen bond between the phenolic -OH group of Tyr108 and the water/hydroxide ligand of the T2 copper can result in redistribution of the electron spin density on the coordinating histidine ligands. Figure 3d shows that the N δ 1 of resonances 13 and 16 have the highest chemical shift perturbation of ∼ - 16 and - 14 ppm, respectively. Interestingly, resonance 13 is in an exchange process with resonance 12 (Fig. 3b) (Dasgupta et al., 2020a), and for the latter resonance the N δ 1 exhibits a downfield shift due to the Y108F mutation. In crystal structure 3cg8 (resolution 2.63 Å), the N δ 1 of His102 from the T2 site can have two hydrogen bonding partners, the carbonyl oxygen of Asp113 and a water molecule (Fig. 4a). Modelling the protons and changing the χ 2 dihedral angle of His102 to - 152 and - 94 ${}^{\circ }$ , hydrogen bonds can be formed between H δ 1–Asp113 CO and H δ 1–H ${}_{2}$ O, respectively. The χ 2 dihedral change does not break the coordination of His102 N ε 2 to the copper (Fig. 4b and c) and is within the allowed range ( - 90 to - 170 ${}^{\circ }$ ) (Dasgupta et al., 2020a). This shows that there can be a conformational exchange of His102 between two states with a hydrogen bond between H δ 1 and either Asp113 CO or the nearby H ${}_{2}$ O molecule. The second-shell mutation of Y108F suggests that the exchanging resonances 13 and 12 are from a H δ 1 nucleus of one of the two T2 copper histidine ligands. Thus, it is proposed that resonances 13 and 12 are from His102 H δ 1, which the ring exchanges between the two states that are shown in panels Fig. 4b and c. Consequently, resonance 16 can be tentatively assigned to the other T2 copper ligand, His234, being also strongly affected by the Y108F mutation. It does not exhibit chemical exchange at temperatures ≤ 298 K, in agreement with having a single, hydrogen bond with Asp259 CO (Fig. 4a). At higher temperatures ( ≥ 303 K), however, exchange with resonance 18 is observed. Whereas the 12/13 pair of resonances shows a difference of less than 3 ppm (Dasgupta et al., 2020a) and a similar linewidth for both signals, the 16/18 pair shows almost 9 ppm difference in chemical shift, and resonance 18 is much broader, indicating a more drastic change in spin density on the proton. In combination with the observation that there are no other hydrogen bond acceptors in the proximity, this suggests that resonance 18 represents the His234 H δ 1 in a state in which the hydrogen bond to Asp259 is broken. In such a state the proton would be prone to exchange with bulk water protons, but the TNC is very buried, preventing rapid exchange. Similar situations to those for His102 are observed for other histidine ligands in the TNC (Table S4). For example, in the crystal structure of SLAC from *S. griseoflavus* PDB entry 6s0o resolution 1.8 Å) (Gabdulkhakov et al., 2019), N δ 1 of His237 can form a hydrogen bond with Asp114 O δ 1 or water O540, depending on rotation around χ 2 (Fig. S5). In the crystal structure of SLAC from *S. coelicolor* (PDB entry 3cg8 resolution 2.68 Å) (Skálová et al., 2009), the equivalent Asp113 O δ 1 is moved away from the N δ 1 and therefore could not form a hydrogen bond (Fig. S5a). Such exchange processes may well represent resonance pairs 3–5 and 9–11. Second-shell mutations around the respective histidine residues can help to confirm this hypothesis.

**Figure 5:**
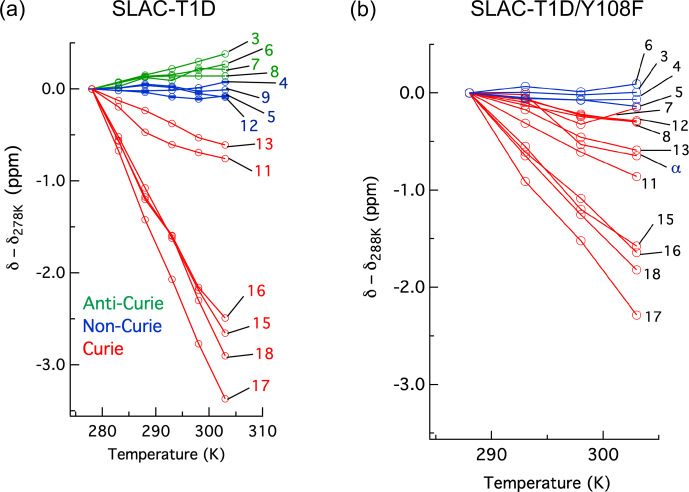
Change in ${}^{1}$ H chemical shifts for **(a)** SLAC-T1D with temperature relative to 278 K and **(b)** SLAC-T1D/Y108F with temperature relative to 288 K. Anti-Curie, non-Curie and Curie behaviours are shown in green, blue and red, respectively.

The temperature dependence of H δ 1 resonances is also affected by the Y108F mutation (Fig. 5). While the resonances that show clear Curie behaviour in SLAC-T1D also do so in the Y108F mutant, resonances that show anti-Curie or non-Curie behaviour tend more to Curie-like behaviour, e.g. resonances 3, 6, 7 and 8. The overall increase in the Curie-like behaviour for the Y108F mutant compared to that of SLAC-T1D can be due to a change in the geometry of the TNC (Solomon et al., 2008) caused by the loss of the hydrogen bond between the Tyr108 and the water/hydroxide.

Slight chemical shift changes are also present for the ${}^{1}$ H resonances between 10 and 20 ppm in the spectrum of SLAC-T1D/Y108F relative to that of SLAC-T1D (Fig. S4). The ${}^{1}$ H– ${}^{1}$ H EXSY/NOESY spectrum shows six cross-peaks (a to f), caused by 12 diagonal signals (Fig. S4). Among these, a1, b1, c1, c2, d1 and e1 are downfield shifted for the mutant, whereas a2, b2, d2 and e2 are upfield shifted (Fig. S4b).

In summary, the Y108F mutation leads to the following tentative assignment of the resonances: 13 and 12 to His102 and 16 to His234 of the T2 site, with 13 and 12 being in chemical exchange.

## 3 Conclusion

The SLAC-T1D comprises resonances from the NI and RO states, in which the RO state is the minor state (Machczynski and Babicz, 2016). Using differently labelled samples and a paramagnetically tailored ${}^{1}$ H– ${}^{15}$ N HMQC experiment, all NI resonances of the N δ 1–H δ 1 groups of the eight coordinating histidine residues in the TNC were accounted for. The HMQC spectra also included the resonances that are in chemical exchange, consistent with the histidine ring motions being responsible for this phenomenon (Dasgupta et al., 2020a). NOE cross-peaks for the RO state revealed resonances of H β protons of the coordinating histidine residues of the T3 site. The second-shell mutation of Y108F of SLAC-T1D aided in the tentative assignment of resonances 13 and 12 to His102 and 16 and 18 to His234 of the T2 site. This report shows the first sequence-specific assignment of the paramagnetically shifted resonance to a coordinating histidine. Clearly, the “blind spot” due to fast nuclear spin relaxation is small for the TNC in the NI state. Potentially, more second-shell residue mutants may help to provide a sequence-specific assignment for all histidine ligands, providing a set of probes to study dynamics in the active site and its possible role in the catalytic mechanism.

## Supplement

10.5194/mr-2-15-2021-supplement The supplement related to this article is available online at: https://doi.org/10.5194/mr-2-15-2021-supplement.

## Data Availability

The original NMR data are uploaded in zenodo.org with https://doi.org/10.5281/zenodo.4392869 (Dasgupta et al., 2020b).
