# Supplementary material for: Towards resolving the complex paramagnetic nuclear magnetic resonance (NMR) spectrum of small laccase: assignments of resonances to residue-specific nuclei
Source: Magn Reson (Gott). 2021 Jan 29;2(1):15–23. doi: 10.5194/mr-2-15-2021 (PMC10539750; doi:10.5194/mr-2-15-2021)
Supplement: The supplement related to this article is available online at: https://doi.org/10.5194/mr-2-15-2021-supplement. [file mr-2-15-supplement.pdf]

Supplement of Magn. Reson., 2, 15–23, 2021
https://doi.org/10.5194/mr-2-15-2021-supplement
© Author(s) 2021. This work is distributed under
the Creative Commons Attribution 4.0 License.

Open Access
MAGNETIC RESONANCE

Supplement of

Towards resolving the complex paramagnetic nuclear magnetic resonance (NMR) spectrum of small laccase: assignments of resonances to residue-specific nuclei

Rubin Dasgupta et al.

Correspondence to: Marcellus Ubbink (m.ubbink@chem.leidenuniv.nl)

The copyright of individual parts of the supplement might differ from the CC BY 4.0 License.

Protein expression and purification

SLAC-T1D and SLAC-T1D/Y108F were expressed and purified as described previously (Dasgupta et al., 2020; Machczynski et al., 2004). For ^{15}N histidine specific perdeuterated labelled sample, 50 mg/L of $^{15}N_3$-L-histidine hydrochloride monohydrate (Sigma Aldrich, USA) was added to the M9 medium consisting of ammonium chloride and D-glucose-1,2,3,4,5,6,6-d7 as nitrogen and carbon sources respectively. A volume of 200 μL of the M9 preculture was transferred to 25 mL of M9 medium prepared in 99.99% D_2O for an overnight preculture, which was used to inoculate 500 mL D_2O-M9 minimal medium. Gene expression and protein harvesting was done as for the uniform ^{15}N labelled sample. Purity was checked by SDS PAGE using a precast Bis-Tris gel (ThermoFischer scientific), as shown in Figure S1a. A band ~ 74 kDa is observed. Under native condition from size exclusion chromatography with multi-angle light scattering (SEC-MALS) the molecular weight of the proteins was ~ 105 kDa, in accord with the expected trimeric form.

NMR spectroscopy

Samples contained ~ 1 mM of protein in 10 mM sodium phosphate buffer pH 7.3 with 10% D_2O. Experiments were done on a Bruker AV-III HD 600 MHz NMR spectrometer equipped with a TXI cryoprobe. 1D ^1H WEFT, Inversion recovery experiments to measure the spin-lattice relaxation rate and 2D ^1H-^1H EXSY/NOESY (Jeener et al., 1979) experiments were recorded as described previously (Dasgupta et al., 2020). The mixing time dependent integral volume profiles were fitted using equation described previously (Dasgupta et al., 2020; Farrow et al., 1994) with Igor Pro 6.3.7 to obtain the exchange rates. The fitting was done by constraining the K_{eq} (Table S1) to the value of the ratio of the diagonal integral volume at 0 ms mixing time. The spin-lattice relaxation rates used in the fitting were obtained from inversion recovery experiments.

2D ^1H-^{15}N HMQC experiments were recorded using the pulse sequence shown in Figure S2. The transfer delay d2 was optimized to 0.5 ms to enhance the paramagnetically shifted signals > 20 ppm. Forty eight t1 increment points were acquired with 30720 number of scans corresponding to the total acquisition time of 54 h for each experiment.

Equation to fit the temperature dependence of the chemical shift for the two-metal center

The observed chemical shift δ_{obs} is given by

$$\delta_{obs} = \delta_{dia} + \frac{A}{\hbar}\frac{g_e \mu_B}{\gamma k_B T}\left[\frac{e^{\left(\frac{2J}{k_B T}\right)}}{1 + 3e^{\left(\frac{2J}{k_B T}\right)}}\right] \tag{S1}$$

where δ_{dia} is the diamagnetic chemical shift, A is the isotropic hyperfine coupling constant, g_e is the free-spin electron *g* factor with the value of 2.0023, μ_B is the electron Bohr magneton, $\hbar$ is the reduced Planck constant, γ is the gyromagnetic ratio of the nucleus in question, k_B is the Boltzmann constant, T is the temperature in Kelvin and $S = 1/2$ which is the spin of the Cu(II) ion (Bertini et al., 2017; Machczynski et al., 2004).

Supporting Figures

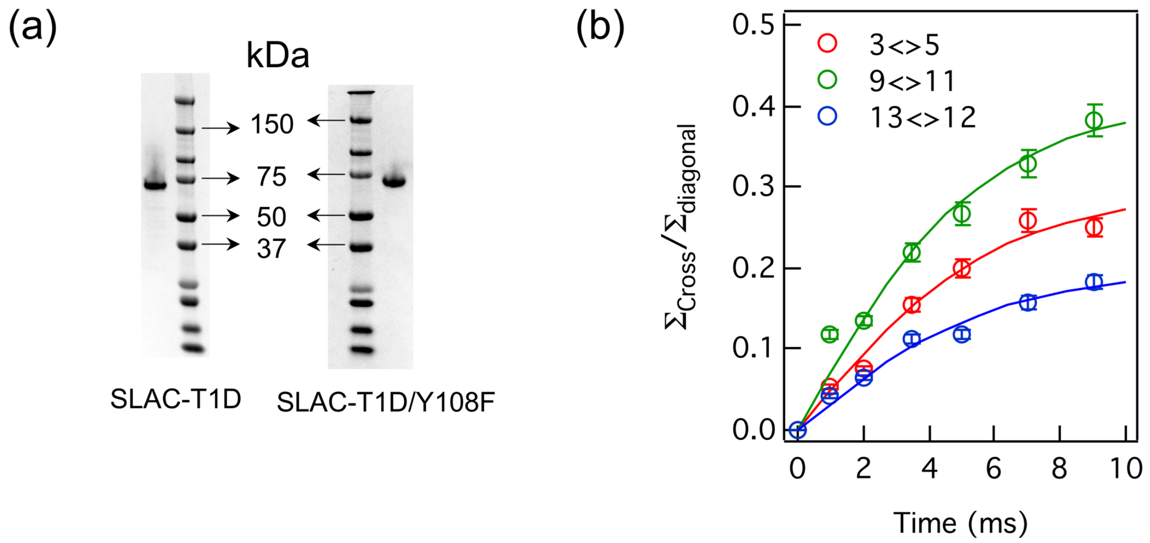

Figure S1: (a) Image of a Bis-Tris precast SDS PAGE gel (ThermoFischer scientific) of purified SLAC-T1D and SLAC-T1D/Y108F; (b) Fits of the intensity profile of normalized cross peak integrals from the resonance pairs of 3-5, 9-11 and 13-12 from 2D ^{1}H-^{1}H EXSY of SLAC-T1D/Y108F to determine the exchange rates (Dasgupta et al., 2020; Farrow et al., 1994). The error bars represent the standard error of the mean derived from the duplicate experiments.

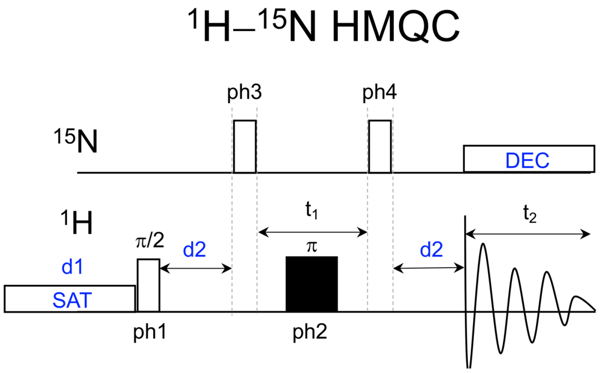

Figure S2: ^{1}H-^{15}N HMQC pulse sequence used in this study. Open rectangles are for 90° pulse and filled ones are for 180° pulse. The interscan delay d1 is 100 ms and SAT is the continuous wave water saturation pulse with phase of x. DEC is GARP composite pulse decoupling during the t_2 evolution. The evolution period d2 was set to 0.5 ms, corresponding to the FWHM of the paramagnetically shifted ^{1}H resonances (~ 0.8 ppm) (Bertini et al., 2017; Ciofi-Baffoni et al., 2014). The phase cycling is ph1 = x, ph2 = x, ph3 = x -x, ph4 = x x -x -x and for the receiver it was x -x x -x.

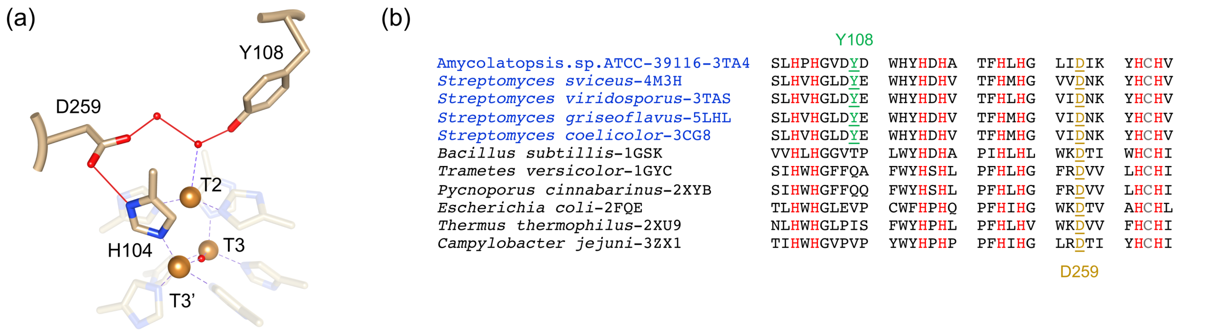

Figure S3. The second coordination shell residue Tyr108. (a) Crystal structure of SLAC (PDB entry 3cg8, from *Streptomyces coelicolor*, resolution 2.68 Å) (Skálová et al., 2009), showing the hydrogen bonding network between Tyr108 and His104 via Asp259 (red lines). The hydrogen bonds were determined by the default parameter of UCSF Chimera program (Pettersen et al., 2004) with relaxed hydrogen bond constraints of 0.4 Å and 20.0° (Mills and Dean, 1996); (b) Sequence alignment of the two-domain (blue) and three-domain laccases (black) for which crystal structures are available (PDB code indicated). The copper coordinating residues are shown in red. Tyr108 and Asp259 (numbering from PDB 3cg8) are shown in green and gold, respectively and are underlined. The type 1 copper site ligand, cysteine, is marked in grey.

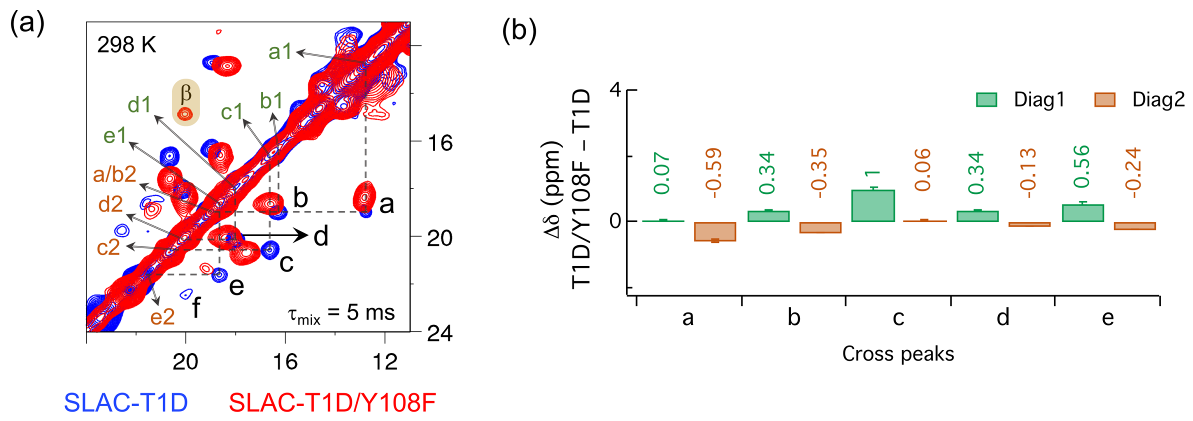

Figure S4. Comparison of the RO spectral regions for SLAC-T1D and SLAC-T1D/Y108F. (a) Overlayed ^{1}H-^{1}H EXSY/NOESY spectra at 298 K with mixing time of 5 ms of SLAC-T1D (blue) and SLAC-T1D/Y108F (red) for the region between 11 and 22 ppm. Cross-peaks and diagonal peaks for SLAC-T1D are marked. The new cross peak β in SLAC-T1D/Y108F is highlighted; (b) Chemical shift changes between SLAC-T1D and SLAC-T1D/Y108F. Positive values mean the diagonal peak in SLAC-T1D/Y108F is downfield shifted, while for the negative value it is upfield shifted.

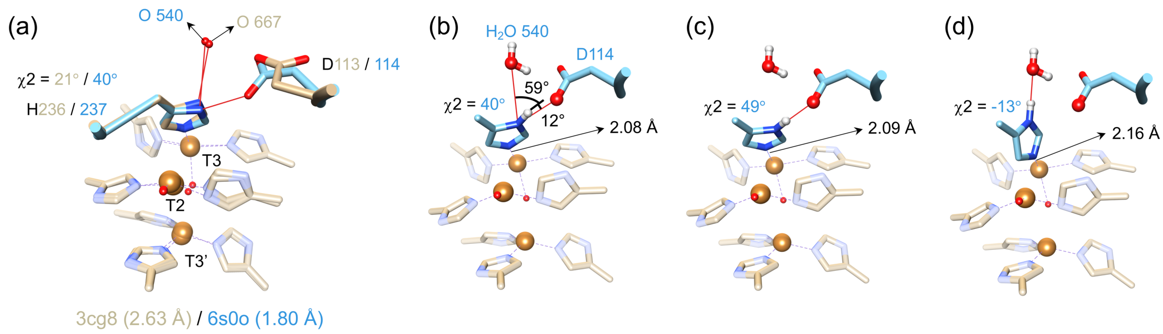

Figure S5. Two H-bond acceptors for His236. (a) Overlayed TNC from crystal structure 3cg8 in gold and 6s0o in blue highlighting the hydrogen bond of the Nδ1 from the T3 histidine ligand His236/237 (Gabdulkhakov et al., 2019; Skálová et al., 2009). The χ2 dihedral angle for histidine 236/237 is shown for both crystal structures. Hydrogen bonds are shown as red lines; (b) Protons are modelled for the crystal structure 6s0o (from *Streptomyces griseoflavus* ; resolution 1.8 Å) using the algorithm as implemented in UCSF Chimera (Gabdulkhakov et al., 2019; Pettersen et al., 2004). The values of the angles between [Asp114 Oδ1 – His237 Nδ1 – His237 Hδ1] and [water O540 – His237 Nδ1 – His237 Hδ1] are indicated along with the distance between His237 Nε2 - T3 copper (with arrows); (c, d) The ring rotation that brings the Hδ1 to the optimal position for a hydrogen bond with Asp114 Oδ1 (c) and the water (d) are shown. The new χ2 dihedral angle and the corresponding His237 Nε2 - T3 copper distances are indicated.

Table S1. Exchange and spin lattice relaxation rates at 298 K for SLAC-T1D/Y108F. In brackets the values for SLAC-T1D are shown for comparison (Dasgupta et al., 2020). Errors in the values are ~ 5% from the duplicate experiments.

	3 – 5	9 – 11	13 – 12
k_A (s^{-1})	37 (33)	51 (29)	26 (29)
k_B (s^{-1})	60 (62)	91 (91)	39 (34)
k_{ex} (s^{-1})	97 (95)	142 (120)	65 (63)
K_{eq} (k_A/k_B)	0.62 (0.53)	0.56 (0.32)	0.67 (0.85)
$R1_A$ (s^{-1})	225 (225)	240 (240)	170 (170)
$R1_B$ (s^{-1})	518 (457)	524 (464)	483 (492)

Table S2. ^{1}H and ^{15}N chemical shift in ppm of the paramagnetically shifted resonances (NI state, > 22 ppm in 1D ^{1}H WEFT spectrum) at 298 K. Resonance showing no change between SLAC-T1D/Y108F and SLAC-T1D are highlighted. Resonance pairs undergoing chemical exchange are color coded as red for 3-5, green for 9-11 and blue for 13-12. ^{15}N chemical shift of resonance 7, 8, 17 and 18 are not observed from the ^{1}H-^{15}N HMQC spectra and are highlighted in grey. Resonance 7 and 8 were reported to be carbon attached protons (Dasgupta et al., 2020) while the ^{1}H resonance of 17 and 18 are broad (~ 1.1 ppm), which can affect the evolution period d2 in the ^{1}H-^{15}N HMQC (Figure S2). Since the d2 was set to 0.5 ms, corresponding to the ^{1}H line with of 0.8 ppm at 600 MHz, resonance 17 and 18 might not be observable due to their larger line broadening.

Resonance	SLAC-T1D		SLAC-T1D/Y108F	
	^{1}H	^{15}N	^{1}H	^{15}N
3	22.00	291.65	22.24	294.24
4	23.40	245.21	24.53	251.16
5	24.75	330.35	25.07	333.41
6	26.46	461.91	25.95	459.73
7	29.26	-	29.26	-
8	31.65	-	31.65	-
9	34.88	450.30	35.43	456.79
11	36.95	477.39	37.68	485.19
12	39.24	535.44	39.20	539.05
13	41.82	581.87	42.04	565.49
15	43.09	569.04	43.13	570.38
16	44.11	574.13	43.67	559.61
17	49.17	-	49.17	-
18	52.52	-	52.52	-

Table S3. ^{1}H and ^{15}N chemical shift in ppm at 298 K for the resonance between 12 and 22 ppm of the RO state.

Resonance	SLAC-T1D	
	^{1}H	^{15}N
a1	12.82	179
a2	19.01	266
b1	16.29	-
b2	19.06	270
c1	16.65	248
c2	20.59	277
d1	18.01	-
d2	20.02	275
e1	18.64	-
e2	21.64	-
x1	13.58	185
x2	14.68	213
y	15.43	232
w	13.29	190
z	19.90	265

Table S4. Potential hydrogen bond acceptors for T3 His Nδ1 atoms as defined by (Mills and Dean, 1996). Data based on chains E and F from PDB entry 6s0O (resolution 1.80Å) (Gabdulkhakov et al., 2019).

Histidine	Potential hydrogen bond acceptors
His 105.E	Asp 260.F Oδ1 and H_2O 546.E O
His 290.F	Gln 292.F Oε1 and H_2O 540.F O
His 159.E	Glu 164.E CO and H_2O 540.E O
His 237.F	Asp 114.E Oδ1 and H_2O 501.E O

Table S5. The estimated hyperfine constant A from the fit in of the chemical shift temperature dependence in Figure 2b of the main text using equation S1

Resonance	Hyperfine coupling constant A (MHz)
a1	0.7 +/- 0.2
b1	2.7 +/- 0.3
c1	1.5 +/- 0.1
d1	3.0 +/- 0.5
a2	2.0 +/- 0.2
b2	2.0 +/- 0.2
c2	2.3 +/- 0.4
d2	2.2 +/- 0.3

Supporting References

Bertini, I., Luchinat, C., Parigi, G. and Ravera, E.: NMR of paramagnetic molecules: applications to metallobiomolecules and models, Second edition., Elsevier, Amsterdam., 2017.

Ciofi-Baffoni, S., Gallo, A., Muzzioli, R. and Piccioli, M.: The IR-^{15}N-HSQC-AP experiment: a new tool for NMR spectroscopy of paramagnetic molecules, Journal of Biomolecular NMR, 58(2), 123–128, doi:10.1007/s10858-013-9810-2, 2014.

Dasgupta, R., Gupta, K. B. S. S., Nami, F., Groot, H. J. M. de, Canters, G. W., Groenen, E. J. J. and Ubbink, M.: Chemical Exchange at the Trinuclear Copper Center of Small Laccase from *Streptomyces coelicolor*, Biophysical Journal, 119(1), 9–14, doi:10.1016/j.bpj.2020.05.022, 2020.

Farrow, N. A., Zhang, O., Forman-Kay, J. D. and Kay, L. E.: A heteronuclear correlation experiment for simultaneous determination of ^{15}N longitudinal decay and chemical exchange rates of systems in slow equilibrium, J Biomol NMR, 4(5), 727–734, doi:10.1007/BF00404280, 1994.

Gabdulkhakov, A., Kolyadenko, I., Kostareva, O., Mikhaylina, A., Oliveira, P., Tamagnini, P., Lisov, A. and Tishchenko, S.: Investigations of Accessibility of T2/T3 Copper Center of Two-Domain Laccase from *Streptomyces griseoflavus* Ac-993, International Journal of Molecular Sciences, 20(13), 3184, doi:10.3390/ijms20133184, 2019.

Jeener, J., Meier, B. H., Bachmann, P. and Ernst, R. R.: Investigation of exchange processes by two-dimensional NMR spectroscopy, J. Chem. Phys., 71(11), 4546–4553, doi:10.1063/1.438208, 1979.

Machczynski, M. C., Vijgenboom, E., Samyn, B. and Canters, G. W.: Characterization of SLAC: A small laccase from *Streptomyces coelicolor* with unprecedented activity, Protein Science, 13(9), 2388–2397, doi:10.1110/ps.04759104, 2004.

Mills, J. E. J. and Dean, P. M.: Three-dimensional hydrogen-bond geometry and probability information from a crystal survey, J Computer-Aided Mol Des, 10(6), 607–622, doi:10.1007/BF00134183, 1996.

Pettersen, E. F., Goddard, T. D., Huang, C. C., Couch, G. S., Greenblatt, D. M., Meng, E. C. and Ferrin, T. E.: UCSF Chimera—A visualization system for exploratory research and analysis, Journal of Computational Chemistry, 25(13), 1605–1612, doi:10.1002/jcc.20084, 2004.

Skálová, T., Dohnálek, J., Østergaard, L. H., Østergaard, P. R., Kolenko, P., Dušková, J., Štěpánková, A. and Hašek, J.: The Structure of the Small Laccase from *Streptomyces coelicolor* Reveals a Link between Laccases and Nitrite Reductases, Journal of Molecular Biology, 385(4), 1165–1178, doi:10.1016/j.jmb.2008.11.024, 2009.
